# Anti-fatigue and anti-oxidant effects of curcumin supplementation in exhaustive swimming mice *via* Nrf2/Keap1 signal pathway

**DOI:** 10.1016/j.crfs.2022.07.006

**Published:** 2022-07-16

**Authors:** Yong Chen, Jiajun Wang, Ziheng Jing, Jose M. Ordovas, Jing Wang, Lirong Shen

**Affiliations:** a College of Biosystems Engineering and Food Science, Zhejiang University, Hangzhou, Zhejiang, China; b School of Food Science and Biotechnology, Zhejiang Gongshang University, Xiasha, Hangzhou, 310018, Zhejiang, China; c Hangzhou Beewords Apiculture Co. Ltd., Hangzhou, China; d Henan ZhongdaHengyuan Biotechnology Co. Ltd., Luohe, China; e Human Nutrition Research Center on Aging at Tufts University, Boston, United States; f Ningbo Research Institute, Zhejiang University, Ningbo, Zhejiang, China

**Keywords:** Turmeric extract, Nrf2/Keap1 signaling, Exercise performance, Anti-oxidant defenses, Energy metabolism

## Abstract

Demands for dietary supplements with anti-fatigue effects are growing fast due to increasing societal demands. Moreover, in highly physically active individuals, there are also significant needs for supplements to improve exercise performance. The present study evaluated the potential anti-fatigue and anti-oxidant effects of curcumin in mice using exhaustive swimming test. Male C57BL/6J mice were randomized into six groups: blank control (Rest), swimming control (Con), Vitamin C (Vc), low-dose curcumin (C_50_), middle-dose curcumin (C_100_), and high-dose curcumin (C_200_). After a 4-week intervention, the mice in all groups except the Rest group were subject to an exhaustive swimming test. Then, mice were sacrificed to examine serum biochemical markers and fatigue-related enzymes. Moreover, the gene and protein expressions of signal transduction factors involved in the Nrf2/Keap1 signaling pathway were measured. The results indicated that curcumin significantly enhanced the exercise tolerance of mice in the exhaustive swimming test. Particularly, the swimming time of mice in the C_100_ group was increased by 273.5% when compared to that of mice in the Con group. The levels of blood urea nitrogen, blood ammonia, lactic acid, creatine kinase and lactate dehydrogenase in the C_100_ group were decreased by 13.3%, 21.0%, 18.6%, 16.7% and 21.9%, respectively, when compared to those of mice in the Con group. Curcumin alleviated exercise-induced oxidative stress and significantly enhanced the activities of superoxide dismutase, catalase and glutathione peroxidase by activating the Nrf2 signaling. These findings indicated that curcumin supplementation exerted remarkable anti-oxidant and anti-fatigue effects in mice, providing additional evidence supporting the use of curcumin as functional food, especially by those engaged in sports-related activities.

## Highlights

• Curcumin exerted remarkable anti-oxidant and anti-fatigue effects in mice.
• Curcumin can activate anti-oxidant response *via* Nrf2/Keap1 signaling pathway.
• Curcumin greatly enhanced the exercise tolerance of mice in exhaustive swimming test.
• Curcumin alleviated exercise-induced oxidative stress by its anti-oxidant effects.
• Curcumin can be an anti-fatigue promising candidate improving exercise performance.

## Graphical abstract

**Figure:**
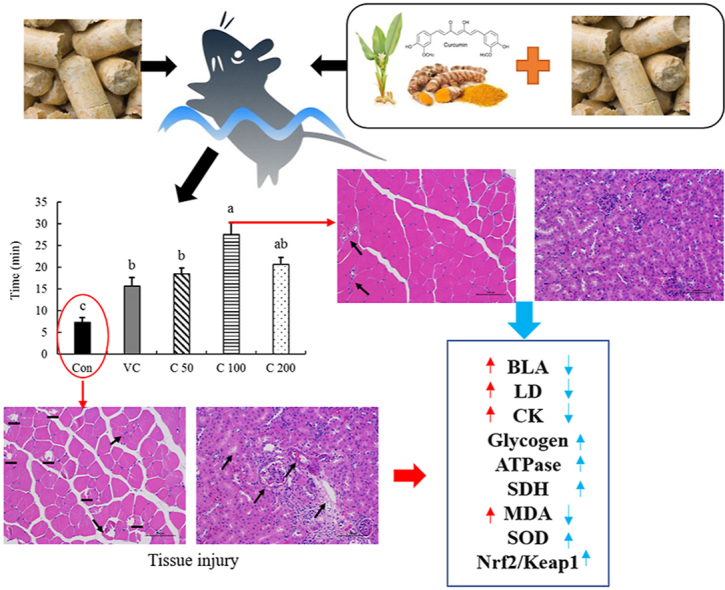

## Abbreviations

ALT: Alanine aminotransferase
ARE: Antioxidant response element
AST: Aspartate aminotransferase
BA: Blood ammonia
BSA: Bovine serum albumin
BUN: Blood urea nitrogen
GLU: Blood glucose
CAT: Catalase
CK: Creatine kinase
CMC: Carboxymethylcellulose
EST: exhaustive swimming test
GSH-Px: Glutathione peroxidase
HG: Hepatic glycogen
HO-1: Heme oxygenase-1
LD: Lactic acid
LDH: Lactate dehydrogenase
MDA: Malondialdehyde
MDH: Malate dehydrogenase
NCDs: Noncommunicable diseases
Nrf2: Nuclear factor E2-related factor 2
NQO1: NAD(P)H: quinine oxidoreductase 1
PK: Pyruvate kinase
qPCR: Quantitative real-time PCR
ROS: Reactive oxygen species
SDH: Succinate dehydrogenase
SOD: Superoxide dismutase
TP: Total protein
WST: Weight-loaded swimming test

## 1 Introduction

Nowadays, chronic diseases such as cardiovascular disease, cancer, obesity and diabetes have become major factors for global morbidity and mortality (Alageel et al., 2017; Marunaka et al., 2020; Stoner, 2020). Physical inactivity contributes 12%–19% to the risks associated with the 5 major NCDs in China (Zhang and Chaaban, 2013). Regular physical activity helps to improve physical and mental functions as well as reverse some effects of chronic diseases. The health benefits of physical activity for improving NCDs has drawn more attentions. With increasing people's awareness of health, regular sports training is becoming more and more popular. As a result, improving exercise performance and reducing exercise-induced muscle damage in physically active are frequent reasons for seeking sport nutrition and dietary supplements (Chen et al., 2020b). Functional foods could no only provide health-promoting effects beyond basic nutrition, but also offer the prevention or supplementary treatment of chronic diseases (Peng et al., 2020; Xie et al., 2019).

Many phytonutrients have been shown to activate nuclear factor E2-related factor 2 (Nrf2) and this process could occur by modifications of cysteine residues which are different from those targeted through exercise (Chen et al., 2022; Jia et al., 2018; Pala et al., 2016). The Nrf2 signaling pathway is involved in many key genes, and the interaction between Nrf2 and Keap1 is highly conserved across species, indicating its important regulatory role (Kobayashi et al., 2002). Nrf2 usually is present in the cytoplasm and binds to Keap1 as a redox-sensitive master regulatory transcriptional factor. Nrf2 dissociates from Keap1 and then binds to ARE in the promoters of phase II anti-oxidant enzyme genes. HO-1 and NQO1 are the most important downstream targets of the Nrf2/Keap1 signal pathway. In addition, NAD(P)H: quinine oxidoreductase 1 (NQO1) and heme oxygenase-1 (HO-1), also known as glutamate-cysteine ligase, play important roles in Nrf2 signaling. NQO1 is a flavor protein that catalyzes reductive metabolic detoxification of redox cycling quinones (Surh, 2021). HO-1 catalyzes the first and rate-limiting step in heme catabolism. Therefore, the above Nrf2 signaling-related genes are primary candidates to study the effects of phytonutrients on exercise performance.

Turmeric (*Curcuma longa* L.) is a yellow-colored rhizomatous herbaceous perennial plant of the Zingiberaceae family, containing turmerin, essential oils, and curcuminoids. Curcumin with the molecular structure composition of (1,7-bis-(4-hydroxy-3-methoxyphenyl)-1,6-heptadiene-3,5 -dione) is the principal curcuminoid of turmeric and a primary bioactive phytochemical that has been used widely as a dietary supplement (Wang et al., 2021; Sun et al., 2018). Epidemiological studies have consistently confirmed the beneficial effects of dietary curcumin on certain aspects of physiological functions, such as preventing fatigue and muscle damage by its anti-oxidant and anti-inflammatory activities (Boz et al., 2015; Huang et al., 2015; Sahin et al., 2016). Moreover, increasing evidence supports that the anti-oxidant activity of curcumin is highly associated with its regulating ability in the Nrf2 signaling pathway (Peng et al., 2018; Soetikno et al., 2013). Numerous studies suggested that curcumin exerts anti-oxidant activity, but few studies have reported that curcumin could improve exercise-induced fatigue by activating the Nrf2 pathway (Huang et al., 2015; Wafi et al., 2019). Even though the anti-oxidant properties of curcumin might significantly contribute to its anti-fatigue effect, the regulation of curcumin on a fatigue-related enzyme activity could also play a critical role in ameliorating exercise-induced fatigue. Specifically, the regulation of curcumin on the fatigue-related enzymes involved in the glycolytic pathway is not well understood. Therefore, this study aimed to investigate the anti-oxidant and anti-fatigue activities of curcumin in mice challenged using exhaustive swimming test and to examine its potential regulation of the glycolytic and Nrf2/Keap1 signaling pathways.

## 2 Materials and methods

### 2.1 Materials and reagents

Curcumin with purity over 99% was obtained from Shanghai Winherb Medical Science Co., Ltd (Shanghai, China). Carboxymethylcellulose (CMC) was purchased from Aladdin Reagent Co., Ltd. (Shanghai, China). The following kits, hepatic glycogen, muscle glycogen, pyruvate kinase (PK), succinate dehydrogenase (SDH), malate dehydrogenase (MDH), Na^+^-K^+^-ATPase, Ca^2+^-Mg^2+^-ATPase, malondialdehyde (MDA), superoxide dismutase (SOD), catalase (CAT)and glutathione peroxidase (GSH-Px) were all purchased from Nanjing Jiancheng Bioengineering Institute (Nanjing, China). All other reagents were of analytical grade and stored according to the instructions.

### 2.2 Animals and experimental protocols

A total of 48 adult male C57BL/6J mice (6 weeks old, weight 18–20 g; SLAC Laboratory Animal Co., Ltd., Shanghai) were housed in the Animal Experiments Center of Zhejiang University under the controlled conditions (temperature of 22 °C ± 1 °C, a 12-h light/12-h dark cycle and humidity of 55% ± 5%). All the animals were treated strictly according to the recommendations of the Guide for the Care and Use of Laboratory Animals of the Science and Technology Commission of P.R.C. (STCC Publication No. 2, revised 1988). The protocol with the ethical approval code of 12628 was approved by the Committee on the Ethics of Animal Experiments Center of Zhejiang University.

After acclimatization for one week, mice were randomized into six groups (n = 8): blank control (Rest), swimming control (Con), Vitamin C (Vc), low-dose curcumin (C_50_), middle-dose curcumin (C_100_), and high-dose curcumin (C_200_). Vc group was administered Vc orally with 40 mg kg^−1^·BW/day for four weeks. The three curcumin groups were fed daily by gavage, 50, 100, and 200 mg curcumin/kg·BW/day for 4 weeks, respectively. Vc and curcumin were weighed and dissolved to the appropriate concentration with 0.3% CMC solution to ensure that the gavage volume was about 0.1 mL. Also, the mice in the Rest and Con group were given the same volume of CMC solution by gavage every day. During the 4-week experimental period, the weights of mice were recorded every week and the weight changes were calculated according to the initial weight and final weight.

### 2.3 Weight-loaded swimming test

The weight-loaded swimming test (WST) was carried out according to the previous studies with mild modifications (Hu et al., 2020; Huang et al., 2015). Briefly, 30 min after the last oral administration, a lead sheath, weighing 5% of their corresponding BW, was fixed to the root of the mouse tail. Then, the mice in all groups except the Rest group were subjected to swim individually in a plastic pool (50 × 50 × 40 cm) filled with water (25 ± 1 °C) to a depth of 30 cm. The weight-loaded swimming time was recorded when the mice sank into the water and failed to rise to the surface for breath within a period of 10 s. During the swimming test, a glass rod was used for stirring gently to make the water in the pool moving continuously and to keep the mice swimming to exhaustion. Then, the mice were removed from the water and dried immediately. After resting for 60 min, the mice were fully anesthetized by intraperitoneal injection of 2% pentobarbital sodium (40 mg kg^−1^) and sacrificed by cervical dislocation to collect the whole blood and tissues for further analyses. All these experimental and care procedures used for animal euthanasia were performed in accordance with the relevant guidelines and regulations, and approved by the Committee on the Ethics of Animal Experiments Center of Zhejiang University. After dissection, the tissues were weighed to calculate the organ indexes according to the formula as follows:

$$Organ\;indexes\;\left(\%\right)=100\text{*}\frac{\text{Organ weight}}{\text{Final body weight}}$$

### 2.4 Histological analysis

The skeletal muscles and kidney tissues were harvested for histopathologic evaluation. Histological examination was done according to the previous studies (Chen et al., 2020b; Soetikno et al., 2013). Briefly, formalin (10%) fixed the tissues were processed for dehydration. After dehydration, the tissues were embedded in paraffin and 4 μm thick sections were cut. Then, the sections were rehydrated through a graded series of ethanol solutions after removing wax by xylene. Hydrated sections were stained with hematoxylin & eosin and examined using light microscopy. Histological scoring for tissues according to an injury grading score system (Peng et al., 2018)with some modifications (Grade 0–4: Grade 0, no pathological change; Grade 1, presence of rare foci of necrosis; Grade 2, small area of mild necrosis; Grade 3, area of mild necrosis severer than Grade 2; and Grade 4, the necrosis severer than Grade 3), was performed by pathologists from Hangzhou Huashu Biotechnology Co., Ltd.

### 2.5 Determination of serum biochemical parameters

Serum was separated from whole blood after centrifugation at 3000 rpm for 10 min at 4 °C. The serum levels of alanine aminotransferase (ALT), aspartate aminotransferase (AST), total protein (TP), blood glucose (GLU), blood urea nitrogen (BUN), blood ammonia (BA), lactic acid (LD), lactate dehydrogenase (LDH) and creatine kinase (CK) were measured by using a 3100 automatic biochemistry analyzer (Hitachi Ltd., Tokyo, Japan).

### 2.6 Examination of hepatic glycogen and muscular glycogen content

After dissecting the mice, part of the liver and muscle tissues were taken and weighed for glycogen content analysis. The hepatic glycogen and muscular glycogen analysis were estimated by commercial assay kits according to the manufacturer's guide.

### 2.7 Analysis of enzyme levels related to energy metabolism anti-oxidant activity in liver tissue

The liver tissues of mice were crushed with the appropriate amount of normal saline, centrifuged at 3000 rpm for 10 min, and the supernatant was used to measure the activities of PK, SDH, Na^+^-K^+^-ATPase, and Ca^2+^-Mg^2+^-ATPase levels by the commercial kits according to the instructions. The malondialdehyde content and anti-oxidant enzyme levels in the livers of mice, including MDA, GSH-Px, CAT and SOD, were estimated by the commercial kits according to the vendor's instructions.

### 2.8 Quantitative real-time PCR (qPCR)

The related genes of the *Nrf2/Keap1* signal pathway, including *Nrf2*, *Keap1*, *HO-1* and *NQO1*, were selected for qPCR analysis and the primers (Table 1) were synthesized by TaKaRa. The method of qPCR was performed as described previously (Chen et al., 2020b). Briefly, total RNA was extracted from each sample (∼100 mg liver tissues) by using RNAiso Plus (TaKaRa) and then was synthesized into cDNA with a PrimeScrit RT reagent Kit (TaKaRa) according to the manufacturer's protocol. The purity and concentration of RNA were determined by measuring the ratio at 260/280 nm using a nanodrop (Thermo Scientific, USA) spectrometer- BioDropμLite (Biodrop, UK). The ViiA™ 7 Real-Time PCR system with fast 96-well block and SYBR Premix Ex Taq (TliRNaseH Plus) (TaKaRa) were used in the qPCR analysis. Cycling conditions were 95 °C for 5 min followed by 40 repeated cycles of 95 °C for 10 s and 60 °C for 30 s. The mRNA expression was determined by comparison with those of the control sample after normalization to *GAPDH* levels and calculated by using the 2^–ΔΔCt^ method. All the analyses were repeated six times.

**Table 1:** Primer sequences used for quantitative real-time PCR (q-PCR).

Gene	Primer sequences	
*GAPDH*	Forward (5′-3′)	CGTGCCGCCTGGAGAAACC
	Reverse (5′-3′)	TGGAAGAGTGGGAGTTGCTGTTG
*Nrf2*	Forward (5′-3′)	TCTCCTCGCTGGAAAAAGAA
	Reverse (5′-3′)	AATGTGCTGGCTGTGCTTTA
*HO-1*	Forward (5′-3′)	CCTCACTGGCAGGAAATCATC
	Reverse (5′-3′)	CCTCGTGGAGACGCTTTACATA
*Keap1*	Forward (5′-3′)	AAGGACCTTGTGGAAGACCA
	Reverse (5′-3′)	CCTGTCCACTGGAATTGAT
*NQO1*	Forward (5′-3′)	TATCCTTCCGAGTCATCTCTAGCA
	Reverse (5′-3′)	TCTGCAGCTTCCAGCTTCTTG

### 2.9 Western blotting

Western blot analyses were performed as described previously (Chen et al., 2020b). Primary antibodies for Nrf2, Keap1, HO-1 and NQO1 (1:1000) were incubated overnight, followed by incubation with secondary antibodies for 1 h (goat anti-rabbit 1:5000 dilution for both Nrf2, Keap1, HO-1 and NQO1). Antibodies of Nrf2, Keap1, HO-1, NQO1 and GAPDH were purchased from Abcam (Abcam, Cambridge, MA, USA). Bands were detected by the ECL Western blot detection reagents (Thermo Fisher Scientific, Waltham, MA, USA) and exposed to a Mini-Protean 3 System (Bio-Rad, Atlanta, GA, USA). Protein band intensities were normalized to GAPDH band intensities using the Image J software. All experiments were performed in triplicate.

### 2.10 Statistical analysis

Statistical analyses were performed using SPSS 19.0 (Statistical Package for the Social Sciences software, SPSS Inc., Chicago, USA). To evaluate the difference among groups, data were analyzed by using one-way ANOVA with Duncan's new multiple range test at *p* < 0.05. Data are presented as the mean ± standard error (SE) for at least three replicates for each sample (Xin et al., 2016).

## 3 Results

### 3.1 Effect of curcumin supplementation on body weight and organ indexes in mice

After the 4-week intervention, there were no significant differences in the body weights among the Rest group (Rest), Control group (Con), Vitamin C group (Vc), and three doses of curcumin groups (C_50_, C_100_ and C_200_) (*P* > 0.05) (Table 2). Also, there were no significant differences in the organ indexes of the heart, kidney and spleen in mice, whereas the liver index of Con was significantly higher than the others. In addition, the organ indexes of testis and epididymis of mice in C_200_ were comparatively lower than the other groups of the mice.

**Table 2:** Effect of curcumin supplementation on body weight and organ indexes in mice.

Body weight (g)	Rest	Con	V_C_	C_50_	C_100_	C_200_
Initial weight	22.38 ± 0.68	21.44 ± 1.15	22.95 ± 0.85	21.73 ± 0.50	21.53 ± 0.64	21.70 ± 0.90
Final weight	24.20 ± 1.03	23.79 ± 1.05	24.64 ± 1.43	24.42 ± 1.35	22.95 ± 1.04	24.24 ± 0.88
Weight gain	1.82 ± 0.68	2.35 ± 0.56	1.69 ± 1.29	2.69 ± 1.31	1.42 ± 0.67	2.54 ± 0.31
**Organ indexes (%)**						
Heart	0.69 ± 0.16	0.67 ± 0.11	0.62 ± 0.09	0.70 ± 0.15	0.69 ± 0.07	0.64 ± 0.09
Liver	3.91 ± 0.46^b^	5.04 ± 0.55^a^	4.05 ± 0.37^b^	4.05 ± 0.35^b^	3.91 ± 0.39^b^	3.84 ± 0.31^b^
Kidney	1.17 ± 0.07	1.14 ± 0.10	1.13 ± 0.07	1.14 ± 0.07	1.15 ± 0.05	1.20 ± 0.11
Spleen	0.26 ± 0.02	0.27 ± 0.05	0.26 ± 0.03	0.26 ± 0.03	0.27 ± 0.03	0.27 ± 0.03
Testis	0.73 ± 0.08^ab^	0.71 ± 0.06^ab^	0.69 ± 0.07^ab^	0.74 ± 0.06^a^	0.72 ± 0.20^ab^	0.57 ± 0.26^b^
Epididymis	0.14 ± 0.02^a^	0.16 ± 0.03^a^	0.14 ± 0.03^a^	0.15 ± 0.02^a^	0.16 ± 0.02^a^	0.11 ± 0.04^b^

Note: Differences were analyzed with one-way ANOVA and Duncan's new multiple range test. ^a–b^ Different letters mean significant difference (P < 0.05). Data are expressed as mean ± S.E.M. (n = 6). The abbreviation of groups used in the figure were: blank control (Rest), swimming control (Con), Vitamin C (Vc), low-dose curcumin (C_50_), middle-dose curcumin (C_100_), and high-dose curcumin (C_200_).

### 3.2 Effect of curcumin supplementation on the weight-loaded swimming capacity

**Fig. 1:**
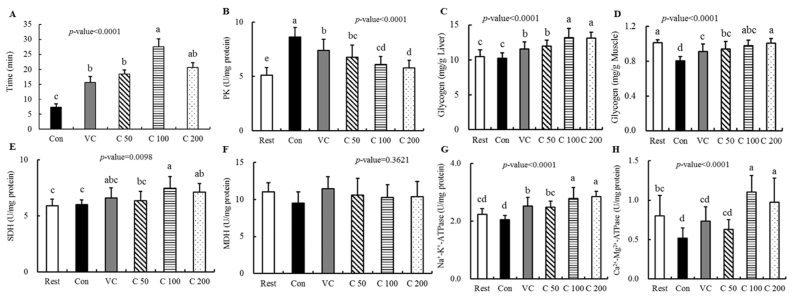
Effect of curcumin supplementation on the weight-loaded swimming capacity (A) and energy metabolic enzymes. (B) Pyruvate kinase, PK; (C) Hepatic glycogen; (D) Muscle glycogen; (E) Succinate dehydrogenase, SDH; (F) Malate dehydrogenase, MDH; (G) Na^+^-K^+^-ATPase; (G) Ca^2+^-Mg^2+^-ATPase. Differences were analyzed with one-way ANOVA and Duncan's new multiple range test. ^a–e^ Different letters mean significant difference (P < 0.05). Data are expressed as mean ± S.E.M. (n = 6). The abbreviation of groups used in the figure were: blank control (Rest), swimming control (Con), Vitamin C (Vc), low-dose curcumin (C_50_), middle-dose curcumin (C_100_), and high-dose curcumin (C_200_).

As shown in Fig. 1-A, the weight-loaded swimming time of mice in Vc and curcumin supplementation groups was significantly higher than that of mice in the Con group (*P*＜0.0001), which indicated that curcumin supplementation could markedly reinforce the exercise tolerance and ease the degree of physical fatigue of mice. Particularly, the weight-loaded swimming time of mice in the C_100_ group was increased by 273.5%, compared to that of mice in the Con group, and was 1.76 times that of mice in the Vc group.

### 3.3 Effect of curcumin supplementation on the serum biochemical parameters of mice

In order to determine the effect of curcumin supplementation on the metabolism of mice after exhaustive swimming, a total of ten serum biochemical parameters of mice in each group were measured. The results were shown in Table 3. Firstly, there were no significant differences in the total protein (TP) and AST/ALT ratio of all groups. Secondly, compared to that of mice in Rest group, the GLU of mice in Con group was significantly decreased, but no significant differences were found in Vc and curcumin supplementation groups. On the other hand, the ALT, AST, BUN, BA, LA, CK and LDH levels of mice in the Con group were remarkably increased after exhaustive swimming compared to those of mice in the Rest group. Interestingly, these levels were decreased in mice treated with curcumin when compared to those of mice in the Con group. Among all these levels, the levels of BUN, BA, LA, CK and LDH were analyzed to determine the metabolic changes in the anti-fatigue activity of curcumin supplementation. Taking the results of mice in the C_100_ group that showed the best performance in weight-loaded swimming time as an example, we found that the levels of BUN, BA, LA, CK and LDH of mice in the C_100_ group were significantly decreased by 13.3%, 21.0%, 18.6%, 16.7% and 21.9%, compared to those of mice in the Con group, respectively. Thus, the results of serum biochemical parameters indicated that curcumin supplementation could improve the exercise tolerance and remit the degree of physical fatigue of mice by modulating physiological and metabolic changes in fatigue-related factors.

**Table 3:** Effect of curcumin supplementation on the serum biochemical parameters of mice.

Biochemical indexes	Rest	Con	V_C_	C_50_	C_100_	C_200_
ALT (U/L)	56.50 ± 6.63^b^	137.50 ± 15.19^a^	79.75 ± 5.09^b^	77.25 ± 8.95^b^	85.38 ± 6.61^b^	87.5 ± 8.23^b^
AST (U/L)	154.75 ± 15.96^c^	418.75 ± 32.52^a^	245.13 ± 17.82^bc^	241.25 ± 22.21^bc^	277.5 ± 25.16^b^	233.75 ± 24.85^bc^
TP (g/dL)	6.59 ± 0.19	6.58 ± 0.13	6.58 ± 0.08	7.11 ± 0.23	7.11 ± 0.24	7.05 ± 0.24
GLU (mg/dL)	5.79 ± 0.20^a^	4.75 ± 0.26^b^	5.85 ± 0.16^a^	6.45 ± 0.28^a^	6.29 ± 0.35^a^	6.58 ± 0.47^a^
BUN (mmol/L)	4.72 ± 0.27^d^	8.69 ± 0.49^a^	6.03 ± 0.32^c^	6.83 ± 0.35^bc^	7.54 ± 0.28^b^	6.65 ± 0.18^bc^
BA (μmol/L)	77.37 ± 6.13^c^	141.09 ± 9.09^a^	112.06 ± 5.55^ab^	100.90 ± 8.73^bc^	111.42 ± 7.74^ab^	98.05 ± 8.65^bc^
LA (mmol/L)	5.43 ± 0.18^d^	11.77 ± 0.69^a^	8.31 ± 0.34^bc^	7.46 ± 0.28^c^	9.58 ± 0.29^b^	8.79 ± 0.39^bc^
CK (U/L)	455.31 ± 40.99^c^	858.78 ± 36.82^a^	624.49 ± 27.30^b^	630.75 ± 24.30^b^	715.31 ± 32.41^b^	638.65 ± 61.08^b^
LDH (U/L)	456.38 ± 38.43^d^	2327.13 ± 138.10^a^	1274.38 ± 106.90^c^	1051.02 ± 88.09^c^	1816.13 ± 59.42^b^	1462.88 ± 134.16^bc^
AST/ALT	2.99 ± 0.48	3.30 ± 0.47	3.14 ± 0.27	3.31 ± 0.41	3.49 ± 0.52	2.76 ± 0.25

Note: Differences were analyzed with one-way ANOVA and Duncan's new multiple range test. ^a–d^ Different letters mean significant difference (P < 0.05). Data are expressed as mean ± S.E.M. (n = 6). The abbreviation of groups used in the figure were: blank control (Rest), swimming control (Con), Vitamin C (Vc), low-dose curcumin (C_50_), middle-dose curcumin (C_100_), and high-dose curcumin (C_200_).

### 3.4 Effect of curcumin supplementation on hepatic and muscular glycogen levels

The glycogen levels in the liver and skeletal muscle were shown in Fig. 1-C, D. Regarding the hepatic glycogen (HG) level (Fig. 1-C), there were no significant differences between those of mice in the Rest group and Con group. However, compared to that of mice in the Con group, the HG levels of mice after 4-week of Vc or curcumin supplementation were significantly increased (*P*＜0.0001),and the HG levels of mice in the C_100_ group were 1.29 times of that in the Con group. On the other hand, the muscular glycogen levels of mice in the Con, Vc and C_50_ groups were remarkably reduced after exhaustive swimming compared with that of mice in the Rest group (Fig. 1-D). However, compared to that of mice in the Con group, the muscular glycogen levels of mice in all the intervention groups were significantly improved by Vc or curcumin supplementation. Consequently, the results illustrated that curcumin supplementation enhanced the glycogen content of mice during prolonged or high-intensity exercise.

### 3.5 Effect of curcumin supplementation on fatigue-related enzyme activities in liver

For further ascertaining the mechanisms of curcumin supplementation in alleviating physical fatigue, the fatigue-related enzyme activities such as PK (Fig. 1-B), SDH (Fig. 1-E), MDH (Fig. 1-F), Na^+^-K^+^-ATPase(Fig. 1-G) and Ca^2+^-Mg^2+^-ATPase (Fig. 1-H) in the liver of mice were measured. After exhaustive swimming, the activities of PK in mice from all the other groups were significantly raised, compared with that of mice in the Rest group. Nevertheless, the PK activities of mice in the intervention groups were dramatically mitigated by Vc and curcumin supplementation compared with that of mice in the Con group. Additionally, compared to the case of the Rest and Con groups, the SDH activities of mice in the C_100_ and C_200_ groups were significantly increased, while there were no significant differences in that of mice treated with Vc and C_50_. Besides, there were no significant differences in the MDH activities of mice among all the groups (*P* = 0.3621). On the contrary, there were conspicuous differences in the activities of Na^+^-K^+^-ATPase and Ca^2+^-Mg^2+^-ATPase of mice among different groups. The results indicated that curcumin supplementation could significantly reinforce these activities, especially for the mice in C_100_ and C_200_ groups. Overall, our findings demonstrated that curcumin supplementation could prolong exercise tolerance and showed anti-fatigue effects in mice by regulating fatigue-related enzyme activities.

### 3.6 Effect of curcumin supplementation on the morphology of skeletal muscle and kidney in mice

**Fig. 2:**
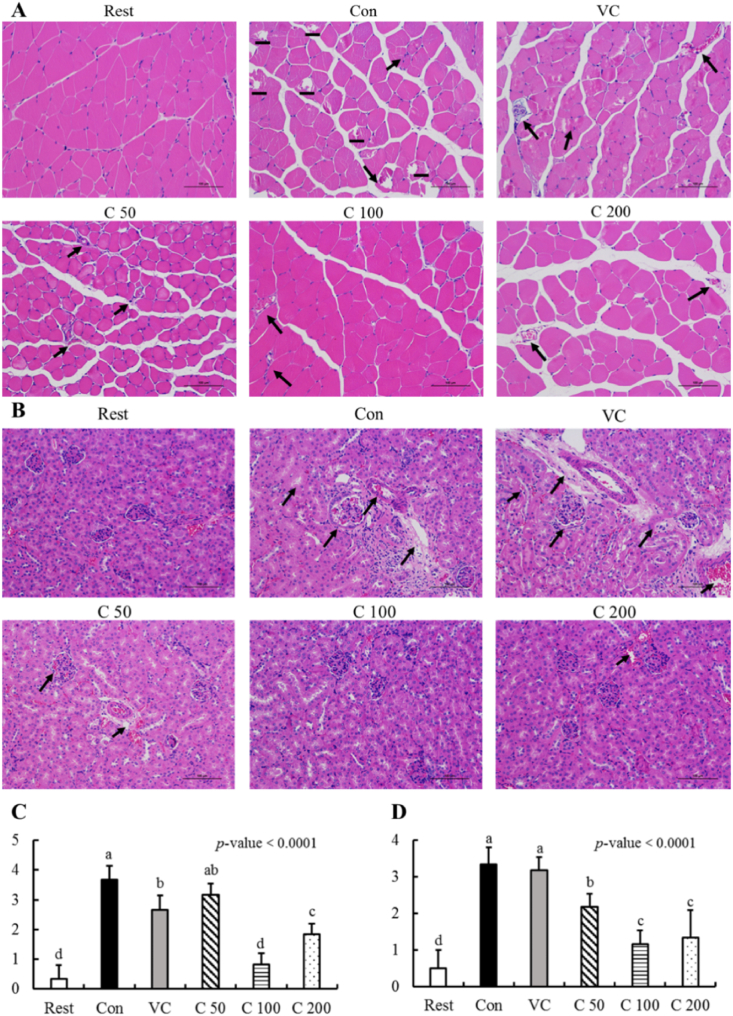
Histological assessmentwith H&E staining (original magnification × 200). (A) The pathological analysis of muscle tissue in mice; (B) The pathological analysis of kidney tissue in mice. Black arrows were used to show the tissue injuries of mice after exhausted swimming stress. Differences were analyzed with one-way ANOVA and Duncan's new multiple range test. ^a–e^ Different letters mean significant difference (*P* < 0.05). Data are expressed as mean ± S.E.M. (n = 6). The abbreviation of groups used in the figure were: blank control (Rest), swimming control (Con), Vitamin C (Vc), low-dose curcumin (C_50_), middle-dose curcumin (C_100_), and high-dose curcumin (C_200_).

After an exhaustive swimming test, the skeletal muscle and kidney in mice were dissected for morphological evaluation. As shown in Fig. 2-A, the skeletal muscle of the Rest group was in a normal muscle tissue structure with neatly arranged muscle fibers, uniform diameter, clear muscle fiber tunica and polygonal cross-section of the muscle fiber (muscle cell). However, after the exhaustive swimming test, the skeletal muscle tissue structure of mice in all the other groups was damaged in varying degrees (just like the lines and arrows in black showed). Compared to the Con group, curcumin supplementation showed protective effects on the skeletal muscle of mice after exhaustive swimming. On the other hand, we also found the same trend in kidney tissue of mice that curcumin supplementation could ameliorate the injuries caused by weight-loaded swimming, especially in the C_100_ group of mice from Fig. 2-B. As a result, there was no distinct difference between the rest group and the C_100_ group. Furthermore, the histological analysis indicated that curcumin supplementation showed significant protective effects compared to the Vc group.

### 3.7 Effect of curcumin supplementation on anti-oxidation related enzyme activities in liver

**Fig. 3:**
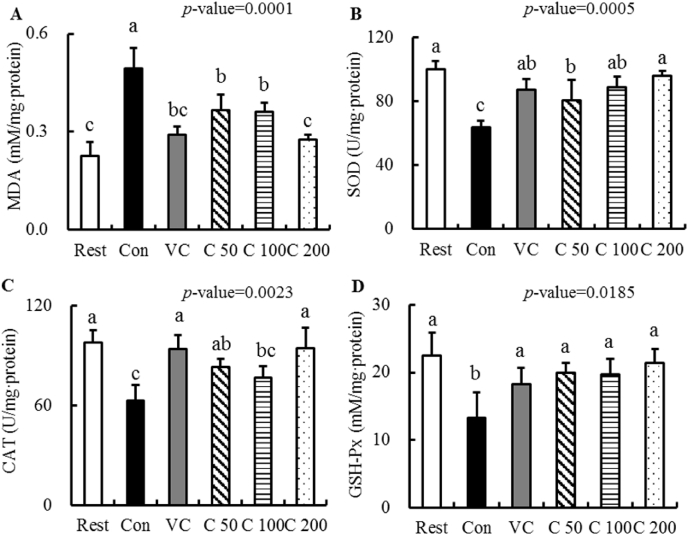
The effect of curcumin on antioxidant enzymes in mice. (A) MDA, (B) SOD, (C) CAT, (D) GSH-Px. Differences were analyzed with one-way ANOVA and Duncan's new multiple range test. ^a–e^ Different letters mean significant difference (*P* < 0.05). Data are expressed as mean ± S.E.M. (n = 6). The abbreviation of groups used in the figure were: blank control (Rest), swimming control (Con), Vitamin C (Vc), low-dose curcumin (C_50_), middle-dose curcumin (C_100_), and high-dose curcumin (C_200_).

To understand the anti-fatigue mechanisms of curcumin supplementation, we examined the anti-oxidation activities in mouse liver in relation to curcumin supplementation. In the present study, the levels of MDA, SOD, CAT and GSH-Px of mouse liver were measured and summarized in Fig. 3. Compared to those of mice in the Rest group, the MDA (Fig. 3-A) level of mice in the Con group was extensively boosted by 119.4%. However, the SOD (Fig. 3-B), CAT (Fig. 3-C) and GSH-Px (Fig. 3-D) levels of mice in the Con group were significantly reduced by 36.7%, 35.6% and 41.2%, respectively. In contrast, compared to those of Con mice, the MDA levels in the intervention groups were significantly decreased and the SOD, CAT and GSH-Px levels were increased. Particularly, there were no significant differences in all the four indexes between the Rest group and C_200_ group, suggesting that curcumin supplementation showed regulation capabilities in anti-oxidation-related enzyme activities of mice during prolonged or high-intensity exercise. These findings provided important clues for clarifying the mechanism of the anti-fatigue activity of curcumin supplementation on exhaustive swimming mice.

### 3.8 Regulation of curcumin supplementation on mRNA and protein expressions of Nrf2/Keap1 signal pathway

**Fig. 4:**
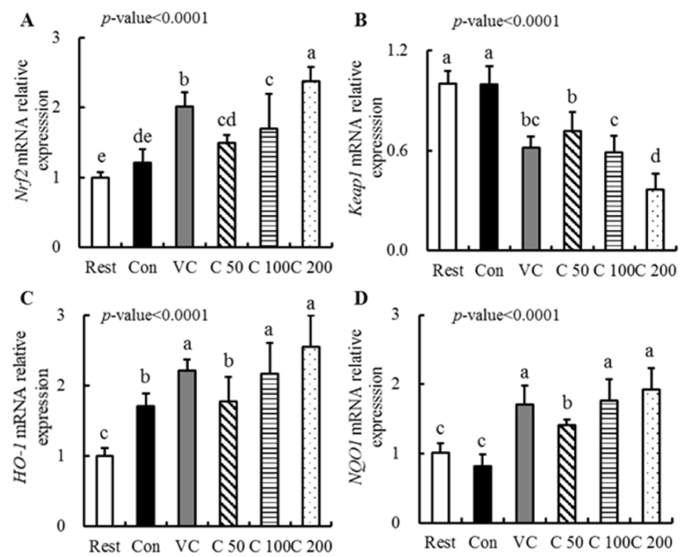
Regulation of curcumin supplementation on mRNA levels in the Nrf2/Keap1 signal pathway.Differences were analyzed with one-way ANOVA and Duncan's new multiple range test. ^a–e^Different letters mean significant difference (P < 0.05). Data are expressed as mean ± S.E.M. (n = 6). The abbreviation of groups used in the figure were: blank control (Rest), swimming control (Con), Vitamin C (Vc), low-dose curcumin (C_50_), middle-dose curcumin (C_100_), and high-dose curcumin (C_200_).

According to the results of anti-oxidation-related enzyme activities in the liver, the mRNA and protein expression of the Nrf2/Keap1 signal pathway were chosen for further study of the regulation mechanism of curcumin in enhancing the anti-oxidant function on mice. The mRNA expressions of *Nrf2* (Fig. 4-A), *Keap1* (Fig. 4-B), *HO-1* (Fig. 4-C) and *NQO1* (Fig. 4-D) were examined and summarized. Among these genes, only the mRNA expression of *HO-1* of mice in the Con group was significantly increased and no significant differences were found in the other three gene expressions compared to the Rest group. However, there were significant differences in all these gene expressions of mice in intervention groups (supplemented by Vc or curcumin) compared to those of mice in the Rest group. Therefore, the results indicated that curcumin supplementation exerted the anti-oxidative and anti-fatigue effects, possibly by regulating related gene mRNA expressions in the Nrf2/Keap1 signal pathway.

**Fig. 5:**
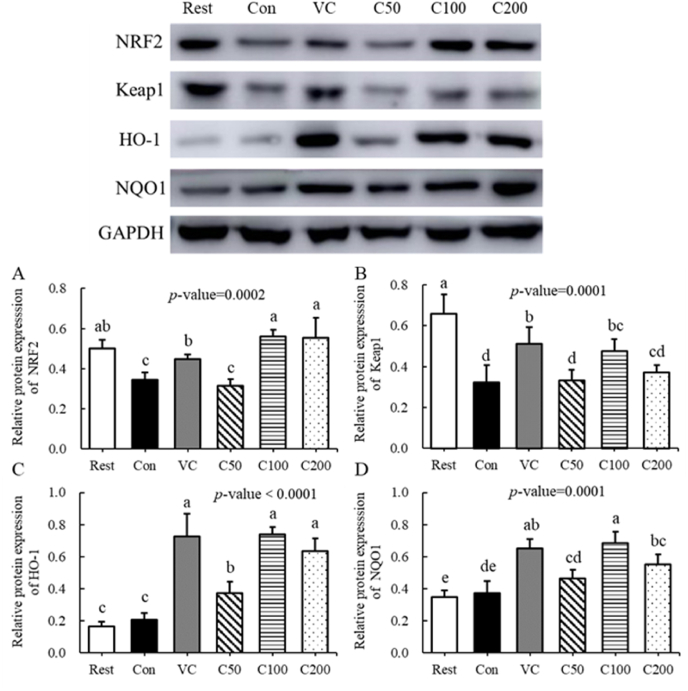
Regulation of curcumin supplementation on the protein expressions in the Nrf2/Keap1 signal pathway. Protein band intensities were normalized to GAPDH band intensities using the Image J software. All experiments were performed in triplicate. Differences were analyzed with one-way ANOVA and Duncan's new multiple range test. ^a–^eDifferent letters mean significant difference (P < 0.05). Data are expressed as mean ± S.E.M. (n = 6). The abbreviation of groups used in the figure were: blank control (Rest), swimming control (Con), Vitamin C (Vc), low-dose curcumin (C_50_), middle-dose curcumin (C_100_), and high-dose curcumin (C_200_).

To further verify the mechanisms of curcumin supplementation in improving the exercise tolerance of mice, the related protein expressions of the Nrf2/Keap1 signal pathway were examined by Western blotting. As presented in Fig. 5, after the weight-loaded swimming test, the protein levels of NRF2 (Fig. 5-A) and Keap1 (Fig. 5-B)of mice in the Con group were significantly decreased and no significant differences were found in protein levels of HO-1 (Fig. 5-C) and NQO1 (Fig. 5-D) when compared to that of mice in the Rest group. However, compared to protein levels in the Con group mice, remarkable increases were found in mice after Vc or curcumin supplementation, especially for the mice in C_100_ group, which had the best performance in the weight-loaded swimming test. Consequently, combining the results of mRNA expressions and protein levels related to the Nrf2/Keap1 signal pathway, it was of great certainty that curcumin supplementation exerted anti-fatigue activities in mice subjected to excessive exercise.

## 4 Discussion

Curcumin, as one of the best known phenolic compounds with numerous medicinal properties and health benefits. However, less information exists about exercise-related effects of curcumin supplementation (Suhett et al., 2021). Moreover, the underlying mechanisms for the improvements of curcumin in physical function remain to be elucidated. Our study demonstrates that curcumin supplementation significantly increases the swimming time of mice probably due to a combination of anti-fatigue and anti-oxidant effects providing preliminary support for its use in the general population and by those engaged in sports-related activities.

Previous studies have shown that LA, BA, CK and LDH levels were closely related to exercise. The high levels of LA formed during high-intensity physical exercise can reduce the pH of muscles and blood, harm certain organs and develop fatigue (Westerblad et al., 2010). In this study, curcumin supplemented groups had lower LA levels, suggesting anti-fatigue activity by preventing LA accumulation and accelerating the LA clearance. On the other hand, ammonia, a fatigue factor was significantly reduced in the curcumin groups. According to previous reports, this decrease of ammonia levels suggests lower muscle catabolism and muscle damage (Chen et al., 2018c). Similarly, LDH is an accurate indicator of muscle activity, whereby elevated serum levels of LDH indicate muscle damage (Huang et al., 2011). Therefore, LDH was identified as an indicator of fatigue in the body and the results demonstrated the anti-fatigue properties of curcumin supplementations by decreasing the LDH levels (Suhett et al., 2021). CK is also a valuable biomarker for skeletal muscle, since it is released during tissue damage, particularly skeletal muscle damage (Westerblad et al., 2010). Prior evidence shows that curcumin supplementation decreased CK activity and muscle damage of rats after eccentric exercise (Boz et al., 2015). In this study, the pathological examination of muscle tissue showed that curcumin supplementation had significant protective effects on the skeletal muscle of mice after exhaustive swimming. Consequently, our data provide evidence that curcumin supplementation regulates fatigue-related biochemical indicators and modulate muscle damage.

Glycogen is a vital energy material that can replenish energy when the blood sugar level is low. Therefore, glycogen plays an essential role in improving exercise tolerance (Xie et al., 2020). During strenuous exercise, hepatic and muscular glycogen are the key sources to satisfy the large amounts of energy consumption. Furthermore, energy metabolism is regulated by various biological enzymes involved in anabolism and catabolism (Greenberg et al., 2006). An exhaustive exercise that may lead to energy metabolism and fatigue imbalance is definitively a very energy-consuming process. The increasing activities of fatigue-related enzymes, such as PK, SDH, MDH, Na^+^-K^+^-ATPase and Ca^2+^-Mg^2+^-ATPase are primary evidence for early fatigue and exercise intolerance. SDH is a key enzyme associated with the regulation of the tricarboxylic acid cycle, catalyzing the synthesis of ATP. In addition, Na^+^-K^+^-ATPase and Ca^2+^-Mg^2+^-ATPase, which play an essential role in the physiological process of material transport, energy conversion, and information transmission, are crucial enzymes to degrade ATP. In the present study, curcumin supplementation remarkably improved the activity of these enzymes to maintain the balance between anabolism and catabolism once the extreme fatigue situation presents through improving the gluconeogenesis of liver energy metabolism in mice subjected to exhausting swimming.

Exhaustive exercise induces a significant increase of reactive oxygen species (ROS) and impairs anti-oxidant defense systems (Banerjee et al., 2003; Ma et al., 2021). Previous evidence has shown that dietary curcumin could enhance anti-oxidant defense systems by activating the Nrf2 signaling pathway, including inhibition of Keap1, affecting the upstream mediators of Nrf2, influencing the expression of Nrf2 and target genes, and finally, improving the nuclear translocation of Nrf2 (Ashrafizadeh et al., 2020; Bellezza et al., 2018). Subsequently, the levels of anti-oxidant enzymes such as SOD, CAT, and GSH-Px are improved apparently by curcumin treatments in many studies (Chen et al., 2020b; Chen et al., 2018a, Chen et al., 2018b; Sahin et al., 2012; Sahin et al., 2016). Furthermore, MDA is one of the final stages of lipid peroxidation products and is an indicator of oxidative stress in cells and tissues, indicating increased membrane damage (Ren et al., 2017). These anti-oxidant enzymes attenuate the increase of MDA induced by exhaustive exercise *via* its ability to reduce lipid peroxidation induced by hydrogen peroxide(Chen et al., 2020a; Xiao et al., 2020). A recent study demonstrated that curcumin could improve the exercise performance of mice in a chronic heart failure model through upregulating Nrf2 and anti-oxidant enzymes (Wafi et al., 2019). In our study, the gene and protein expressions of key factors of Nrf2, Keap1, HO-1 and NQO1, participating in Nrf2/Keap1 signal pathway were examined and confirmed the anti-oxidant modulation mechanism of curcumin on exhaustive exercise mice. As a result, curcumin effectively attenuated oxidative stress and prolonged the exercise tolerance, suggesting that curcumin holds promising potential anti-fatigue function.

To date, several theories, including the “clogging theory”, “exhaustion theory”, “radical theory”, “homeostasis disturbance theory”, “mutation theory” and “protective inhibition theory” have been advanced to interpret the mechanisms of fatigue (Zhong et al., 2017). Among these mechanisms, “exhaustion theory”, “clogging theory” and “radical theory” have attracted more attention (You et al., 2011). The “clogging theory” is concentrated on fatigue-related factors such as LA, BA, CK and LDH levels. The “exhaustion theory” is focused on energy sources like glucose and glycogen. Whereas the well-known “radical theory” suggests the imbalance between the body's oxidation system and its anti-oxidation system (Miao et al., 2018). In recent years, many phytonutrients have shown anti-fatigue effects by reducing exercise-induced oxidative stress (Chang et al., 2022; Zhang et al., 2019; Zhao et al., 2020). In a very early study, it was reported that exhaustive exercise could induce a two-to three-fold increase in free radical concentrations, causing oxidative damage to various tissues and organs (Davies et al., 1982). As a result, the anti-oxidant properties of phytonutrients have been regarded as the dominant hypothesis for interpreting their anti-fatigue effects. Actually, the experimental design of this study was based on the above three key theories to examine the anti-fatigue effects of curcumin on exercise performance. Moreover, the results consistently showed the anti-fatigue and anti-oxidant effects of curcumin in weight-loaded swimming mice.

## 5 Conclusions

In summary, the present study demonstrated the anti-fatigue and anti-oxidant effects of curcumin in a fatigue mice model induced by exhaustive swimming stress. curcumin showed protective effects on exercise-induced fatigue by inhibiting the production of reactive oxygen species. Furthermore, the significant anti-fatigue activity of curcumin worked by efficiently regulating the energy metabolic biomarkers in the glycolytic pathway and activating anti-oxidant response *via* Nrf2/Keap1 signaling pathway. These results suggest that curcumin might be an anti-fatigue promising candidate applied in exercise performance improvement in the future.

## Funding

This work was supported by a grant from the 10.13039/501100004731Natural Science Foundation of Zhejiang Province (LY22C200011), the 10.13039/501100001809National Natural Science Foundation of China (Grant No. 32001692), Ningbo Public welfare science and technology project (2021S029) and the Project of Hangzhou Qianjiang Distinguished Experts (NO. 202038).

## CRediT authorship contribution statement

**Yong Chen:** Conceptualization, Methodology, Validation, Formal analysis, Investigation, Data curation, Writing – original draft, Writing – review & editing. **Jiajun Wang:** Validation, Formal analysis, Investigation, Writing – review & editing. **Ziheng Jing:** Investigation. **Jose M. Ordovas:** Writing – original draft, Writing – review & editing. **Jing Wang:** Supervision, Writing – original draft, Funding acquisition, All authors have read and agreed to the published version of the manuscript. **Lirong Shen:** Conceptualization, Methodology, Validation, Data curation, Writing – original draft, Writing – review & editing, Visualization, Supervision, Project administration, Funding acquisition.

## Declaration of competing interest

The authors declare that they have no known competing financial interests or personal relationships that could have appeared to influence the work reported in this paper.
